# Associations Between Income Status and Perceived Barriers to Using Long-Acting Reversible Contraception: An Exploratory Study

**DOI:** 10.3389/frph.2022.856866

**Published:** 2022-04-12

**Authors:** Leah Henke, Summer Martins, Christy Boraas

**Affiliations:** ^1^Department of Obstetrics and Gynecology, Park Nicollet, Burnsville, MN, United States; ^2^Department of Obstetrics, Gynecology and Women's Health, University of Minnesota Medical School, Minneapolis, MN, United States

**Keywords:** contraception, intrauterine device, survey, United States, barriers

## Abstract

**Introduction:**

Barriers to long-acting reversible contraception (LARC) use in the United States have been described in prior studies, but few have focused on women's income status. We explored associations between income status and perceived LARC barriers in a community-based sample of reproductive-aged women.

**Methods:**

Non-pregnant, heterosexually active women aged 18 to 40 years completed a cross-sectional survey at a large community event in the Midwestern U.S. in 2018. Outcome measures were comprised of 26 survey items gauging perceived barriers to LARC use (e.g., access barriers, side effects). We estimated crude and age-adjusted prevalence ratios (PRs) for each outcome by participants' income status: low-income (≤ 200% of federal poverty guideline) versus higher income.

**Results:**

Low-income women (*n* = 72) were significantly more likely than higher income women (*n* = 183) to endorse 11 of the 26 barriers to LARC use (PR range, 1.23–7.63). Cost of LARC was the most frequently identified access barrier and was more acute for low-income women (PR 1.57, 95% CI 1.17–2.11). After adjustment for age, most associations were attenuated. However, low-income women were still more likely to report concerns about LARC use due to family expectations or beliefs (aPR 3.69, 95% CI 1.15–11.8).

**Conclusion:**

Low-income women perceive more barriers to LARC access and more negative perceptions about use. While these associations also correlate with age, they nonetheless reflect concerns that impact contraceptive equity. Efforts to increase LARC access should address these barriers and focus on concerns more common among low-income women regarding LARC use.

## Introduction

Despite comparable rates of contraceptive use across income levels in the United States, low-income women experience significantly higher rates of unintended pregnancy (1–5). Increasing access to long-acting reversible contraception (LARC) has been identified as a strategy to reduce these disparities. Subdermal upper arm implants and intrauterine contraceptives are classified as LARC and offer high (>99%) efficacy as well as low user requirements (6, 7). In U.S.-based studies, improvements in LARC uptake have been demonstrated with contraceptive counseling, no-cost provision of LARC and post-visit support (8–11). Clinic-based LARC interventions have also correlated with decreases in unintended pregnancy, teen pregnancy and abortions at the population level (2, 9). However, even with interventions to mitigate barriers to LARC uptake, low-income women continue to face an increased risk of unintended pregnancy (2).

Previous studies of U.S. populations have identified numerous barriers to LARC use spanning individual and systemic levels, but few have centered on the experiences of low-income women (10, 12, 13). Data regarding low-income women's pregnancy planning identified themes of lack of reproductive agency, concern about reproductive coercion, and contraceptive use and desire mismatch (14, 15). This secondary analysis explored associations between income status and a broad range of perceived barriers to using LARC in a sample of women attending a large community-based event in the Midwestern U.S.

## Methods

Participants were recruited over three days at the 2018 Minnesota State Fair, a large community event that attracted over 2 million visitors (an estimated 20% of the state population) (16). Inclusion criteria were: female, 18 to 40 years old, English speaking, and not currently pregnant but at risk for pregnancy (vaginal sex with a male partner in the past year). Women were excluded if they were planning a pregnancy in the next 12 months or were biologically incapable of pregnancy due to hysterectomy, bilateral oophorectomy, tubal ligation, or another surgical or medical reason. Participants initially self-screened with posters and hand-outs describing study inclusion and exclusion criteria at the fair's research facility. For fairgoers who expressed interest in participating, eligibility was then confirmed with an electronic survey that terminated if criteria were not met. Electronic surveys were administered anonymously to eligible participants via Qualtrics on iPads or participants' mobile devices using a personalized link. The protocol was deemed exempt by the University of Minnesota institutional review board (STUDY00002291). Participants received a drawstring backpack on completion of the study.

We collected sociodemographic characteristics and reproductive health histories. Using participants' self-reported household income and size, we classified participants as low-income (<200% 2017 federal poverty guideline [FPG]) or higher-income (≥200% FPG). We chose this cut-off due to eligibility through the Minnesota Family Planning Program to cover the costs of contraception for patients below 200% of the FPG (17). Outcome variables were derived from 26 survey items in checklist (yes/no) format gauging perceived barriers to LARC use. Measures were adapted from a previous study of LARC barriers among young homeless women and encompassed 7 access-related barriers (e.g., cost), 11 concerns about side effects, and 8 concerns about other aspects of LARC use (e.g., pain with insertion) (10). For this analysis, we excluded participants with prior LARC use (*n* = 105) or with missing data for age (*n* = 3) or household income (*n* = 5).

We characterized the sample with descriptive statistics and assessed bivariate associations between income group and participant characteristics using Chi-square tests and *t*-tests (α = 0.05). Using modified Poisson regression, we estimated prevalence ratios (PRs) and confidence intervals (CIs) for each outcome variable. We computed both unadjusted and age-adjusted estimates given that income is typically correlated with age, which we also confirmed empirically in our dataset. All analyses were conducted in Stata version 14 (StataCorp, College Station, Texas). Sample size was not determined *a priori* for this analysis.

## Results

During recruitment for the main study, 423 women approached the study team with interest in participation and 365 (86.3%) screened eligible. After excluding current LARC users (*n* = 105) for this analysis, the sample was comprised of 255 survey participants—*n* = 72 (28%) classified as low-income and *n* = 183 as higher income. Overall, the sample was predominantly white and non-Hispanic (Table 1). Compared to higher-income participants, low-income participants were significantly younger (mean age of 25 vs. 30 years), less likely to be married (14 vs. 41%) and had fewer people in their household. They were also less likely to have a higher education degree and have private insurance. There were no significant differences between groups in reproductive health history variables (Table 1).

**Table 1:** Demographic and reproductive health characteristics of participants by income status.

		**Income status**		
**Characteristic**	**Low-income <200% FPG**	**Higher-income ≥200% FPG**	P^*^	
	*n =* 72	*n =* 183		
Age (y)	Mean ± SD	24 ± 6	30 ± 6	<0.01
	18 to 21	32 (44)	15 (8)	<0.01
	22 to 24	17 (24)	20 (11)	
	25 to 29	9 (13)	50 (27)	
	30 to 34	5 (7)	44 (24)	
	31 to 40	9 (13)	54 (30)	
Race/ethnicity	White, non-Hispanic	57 (79)	160 (87)	0.03
	Asian, non-Hispanic	5 (7)	15 (8)	
	Other^†^	10 (14)	8 (4)	
Relationship status	Single	35 (49)	59 (32)	<0.01
	Committed relationship	23 (32)	43 (24)	
	Married	11 (15)	81 (44)	
	Other^‡^	3 (4)	0 (0)	
Vocation	In school	10 (14)	11 (6)	<0.01
	Working	27 (38)	133 (73)	
	Working and in school	30 (42)	27 (15)	
	Other	5 (7)	12 (7)	
Household size	1	47 (65)	56 (31)	<0.01
	2	7 (10)	56 (31)	
	3 or more	18 (25)	71 (39)	
Health insurance type	Public	20 (28)	9 (5)	<0.01
	Private	47 (65)	169 (92)	
	None	5 (7)	5 (3)	
Pregnancies	0	53 (74)	114 (62)	0.15
	1	9 (13)	24 (13)	
	2 or more	10 (14)	45 (25)	
Unintended pregnancies	0	61 (85)	147 (80)	0.62
	1	8 (11)	21 (11)	
	2 or more	3 (4)	15 (8)	
Importance of avoiding pregnancy right now	Very important	54 (75)	119 (65)	0.45
	Somewhat important	10 (14)	32 (17)	
	A little important	4 (6)	20 (11)	
	Not at all important	4 (6)	12 (7)	
Current contraceptive method	Pill	30 (42)	71 (39)	0.84
	Condom	11 (15)	29 (16)	
	Other	16 (22)	50 (27)	
	None	15 (21)	33 (18)	

*FPG, Federal Poverty Guideline (2017); LARC, Long-acting reversible contraception. Data are n (%) unless otherwise specified. Some percentages do not total to 100% due to rounding*.

* *t test, χ2 test, or Fisher's exact test comparing low-income and higher income participants*.

† *Includes Black (3), White and Hispanic (9), Native American (4) and Don't Know (4)*.

‡ *“Other” category excluded from χ2 test*.

Our findings for LARC barriers are detailed in Table 2, in descending order of prevalence among low-income participants. In unadjusted analysis, low-income women were significantly more likely to endorse 4 of 7 perceived barriers related to LARC access: cost, not knowing where to go, family expectations/beliefs, and transportation. Concerns about potential LARC side effects were common and significantly more prevalent among low-income women with respect to weight gain, future fertility, and acne (PR range, 1.23–1.65). Low-income women were also more likely to endorse concerns related to LARC misplacement/displacement, efficacy, and disclosure of use to others. After adjustment for age, associations were attenuated and only family expectations/beliefs remained statistically significant (aPR = 3.69, 95% CI 1.15–11.8).

**Table 2:** Perceived LARC barriers by income status.

	**Income status**		**Prevalence ratio [95% CI]**	
	**Low-income <200% FPG**	**Higher-income** **≥200% FPG**	**Crude**	**Age-adjusted**
	*n =* 72	*n =* 183		
	%	%		
**Perceived barriers to LARC access^†^**				
Cost	54	34	1.57 [1.17–2.11]	1.25 [0.92–1.71]
Need more information	38	26	1.43 [0.97–2.10]	1.10 [0.72–1.67]
Appointment timing	19	13	1.55 [0.84–2.84]	1.48 [0.76–2.88]
Don't know where to go	18	5	3.67 [1.64–8.22]	2.38 [0.93–6.05]
Family expectations or beliefs	11	3	4.07 [1.37–12.0]	3.69 [1.15–11.8]
Transportation	11	4	2.90 [1.09–7.73]	1.88 [0.70–5.07]
Finding childcare	7	4	1.82 [0.59–5.55]	2.30 [0.75–7.05]
**Concerns regarding LARC side effects^†^**				
Weight gain	68	55	1.23 [1.00–1.51]	1.13 [0.90–1.42]
Pain or cramps	54	57	0.95 [0.74–1.22]	0.81 [0.63–1.04]
Problems with future fertility	54	33	1.65 [1.27–2.22]	1.34 [0.97–1.86]
Acne	46	28	1.61 [1.15–2.27]	1.16 [0.80–1.69]
Mood swings	43	42	1.02 [0.75–1.40]	0.89 [0.64–1.25]
Irregular bleeding	42	52	1.21 [0.95–1.55]	1.06 [0.81–1.39]
Risk of cancer	40	36	1.13 [0.80–1.60]	1.04 [0.72–1.50]
Risk of pelvic infections	39	32	1.23 [0.86–1.76]	1.10 [0.75–1.60]
Change in sex drive	36	32	1.14 [0.78–1.66]	1.07 [0.72–1.59]
Headache	32	30	1.08 [0.72–1.62]	0.92 [0.61–1.38]
Risk of an ectopic pregnancy	29	28	1.05 [0.68–1.61]	0.82 [0.53–1.29]
**Concerns regarding LARC use^†^**				
Pain with insertion	71	63	1.13 [0.94–1.36]	1.00 [0.82–1.23]
Misplacement of the device	60	45	1.32 [1.03–1.69]	1.08 [0.83–1.42]
The device not working	56	43	1.30 [1.00–1.70]	1.03 [0.77–1.38]
The possibility of it falling out	51	38	1.34 [1.00–1.80]	1.18 [0.85–1.63]
Having a device inside my body	40	37	1.08 [0.77–1.52]	1.19 [0.83–1.73]
My partner being able to feel it	26	17	1.56 [0.94–2.58]	1.24 [0.73–2.09]
Someone finding out I am using it	8	1	7.63 [1.57–37.0]	2.43 [0.39–15.0]
Incongruence with religious beliefs	3	2	1.27 [0.24–6.81]	1.00 [0.17–5.79]

*FPG, Federal Poverty Guideline (2017); LARC, Long-acting reversible contraception*.

† *Participants could select more than one response*.

## Discussion

Our exploratory study sheds light on psychosocial and structural barriers to LARC use by income level, which have been understudied in prior literature. We examined a wide spectrum of potential barriers and found that low-income women were more likely to cite several including family beliefs, impact on future fertility, and device efficacy. Associations were partially driven by age, as they attenuated upon age adjustment. From previous research on attitudes toward IUDs among young women, similar concerns including misplacement, future infertility, and pain were identified (13). Many of these concerns were derived from anecdotal experiences of friends and acquaintances (13). Similar studies have identified mistrust of information provided by healthcare professionals and concern about reproductive coercion as factors influencing LARC uptake (10, 18, 19). The impact of these factors in relation to contraceptive choice among low-income women are worth evaluating in larger multivariable analyses with greater statistical power.

Ensuring access to LARC is critical, especially for subgroups such as low-income women who bear a larger burden of unintended pregnancy. Between the contraceptive provision of the Affordable Care Act and the Minnesota Family Planning Program, a large proportion of our study population qualified for contraception at no cost. Despite these measures, half of low-income women in our study identified cost as an obstacle. Whether this finding is due to actual or perceived costs in LARC access is unclear due to the complexity of contraceptive insurance coverage. Financial barriers to contraception have been demonstrated in similar studies of hypothetical and actual cost mitigation of LARC (8, 11). Given the large impact of cost on contraceptive choice among low-income women, the perceived financial burden of LARC despite insurance coverage warrants further study.

One strength of our study is the general racial and economic congruence of our sample to Minnesota where 22% of people have a household income under 200% of the FPG and 79% identify as white, non-Hispanic (20, 21). However, Minnesota has a lower poverty rate and less racial diversity than many other states, which may limit the ability to generalize results to more diverse and lower income populations. Our exploratory study is also limited by its sample size, which resulted in some wide confidence intervals and reduced options for multivariable modeling. Adjusting for age also likely controlled for confounding by other sociodemographic characteristics correlated with age, but our estimates could still be affected by residual confounding. Nonetheless, we hope that our findings will prompt further study of perceived barriers to LARC use experienced by low-income women, including the causal pathways by which income status shapes women's LARC perceptions and access. Future research should focus on exploring ways to further understand these barriers and solutions to help alleviate them.

## Data Availability Statement

The raw data supporting the conclusions of this article will be made available by the authors, without undue reservation.

## Ethics Statement

The studies involving human participants were reviewed and deemed exempt from approval by University of Minnesota Institutional Review Board. The patients/participants provided their written informed consent to participate in this study.

## Author Contributions

LH and CB contributed to conception and design of the study. SM performed the statistical analysis. LH wrote the first draft of the manuscript. LH, CB, and SM wrote sections of the manuscript. All authors contributed to manuscript revision, read, and approved the submitted version.

## Funding

Financial support for this study was received from the University of Minnesota Medical School, Department of Obstetrics, Gynecology and Women's Health. This study was presented as a poster at the American College of Obstetricians and Gynecologists Annual Clinical Meeting in April 2020. This was presented virtually due to the COVID-19 pandemic.

## Conflict of Interest

The authors declare that the research was conducted in the absence of any commercial or financial relationships that could be construed as a potential conflict of interest.

## Publisher's Note

All claims expressed in this article are solely those of the authors and do not necessarily represent those of their affiliated organizations, or those of the publisher, the editors and the reviewers. Any product that may be evaluated in this article, or claim that may be made by its manufacturer, is not guaranteed or endorsed by the publisher.
